# Clinical Teaching: An Evidence-based Guide to Best Practices from the Council of Emergency Medicine Residency Directors

**DOI:** 10.5811/westjem.2020.4.46060

**Published:** 2020-07-03

**Authors:** Sreeja Natesan, John Bailitz, Andrew King, Sara M. Krzyzaniak, Sarah K. Kennedy, Albert J. Kim, Richard Byyny, Michael Gottlieb

**Affiliations:** * Duke University, Division of Emergency Medicine, Durham, North Carolina; † Northwestern University, Feinberg School of Medicine, Department of Emergency Medicine, Chicago, Illinois; ‡ The Ohio State University Wexner Medical Center, Department of Emergency Medicine, Columbus, Ohio; § University of Illinois College of Medicine at Peoria/OSF Healthcare, Department of Emergency Medicine, Peoria, Illinois; ¶ Indiana University School of Medicine, Department of Emergency Medicine, Indianapolis, Indiana; || Washington University in Saint Louis School of Medicine, Department of Emergency Medicine, St. Louis, Missouri; # Denver Health Medical Center, Department of Emergency Medicine, Denver, Colorado; ** Rush Medical Center, Department of Emergency medicine, Chicago, Illinois

## Abstract

Clinical teaching is the primary educational tool use to train learners from day one of medical school all the way to the completion of fellowship. However, concerns over time constraints and patient census have led to a decline in bedside teaching. This paper provides a critical review of the literature on clinical teaching with a focus on instructor teaching strategies, clinical teaching models, and suggestions for incorporating technology. Recommendations for instructor-related teaching factors include adequate preparation, awareness of effective teacher attributes, using evidence-based-knowledge dissemination strategies, ensuring good communication, and consideration of environmental factors. Proposed recommendations for potential teaching strategies include the Socratic method, the One-Minute Preceptor model, SNAPPS, ED STAT, teaching scripts, and bedside presentation rounds. Additionally, this article will suggest approaches to incorporating technology into clinical teaching, including just-in-time training, simulation, and telemedical teaching. This paper provides readers with strategies and techniques for improving clinical teaching effectiveness.

## BACKGROUND

Emergency medicine (EM) is a dynamic specialty that requires not only an acquisition of vast amounts of medical knowledge, but also the ability to prioritize and task switch efficiently and effectively to combat the chaos, high patient volume, and variable acuity within a given shift. Additionally, mounting pressures are placed on EM faculty to use less time to care for a larger volume of patients while increasing patient satisfaction scores, documentation, billing, and academic productivity.^1^,^2^ All of these factors can make the emergency department (ED) a challenging environment for clinical teaching.^3^ Moreover, EM faculty concerns over time constraints and patient census variability are reflected in a decline of bedside teaching in the clinical setting.^2^,^4^–^6^ There is mounting evidence that clinician educators often feel ill prepared to teach in this dynamic clinical environment due to a lack of a consolidative resource.^7^–^13^ A set of guidelines may help assist in the development of skills for educators to help bridge this gap.

However, the ED environment provides unique opportunities for clinical teaching due to the breadth of pathology, spectrum of acuity, and large number of clinical encounters. When surveyed, students rated the ED as the most valued rotation for learning opportunities.^14^ Therefore, it is essential that all emergency clinicians who work with learners develop strong clinical teaching skills to maximize this educational opportunity. This article provides a narrative summary of the literature and best practice recommendations for clinical teaching in medical education with a focus on their application within the ED environment.

## CRITICAL APPRAISAL OF THE LITERATURE

This article is the fourth in a series of evidence-based best practice reviews from the Council of Emergency Medicine Residency Directors (CORD) Best Practices Subcommittee.^15^–^17^ With assistance of a medical librarian, we performed a search of Embase, CINAHL, Ovid MEDLINE, and PsycINFO for articles published from inception to April 23, 2018, using keywords and medical subheadings (MeSH) terms focused on teaching at the patient’s bedside. The full search strategy is available in the Appendix. Bibliographies of all relevant articles were reviewed for additional studies. We used social media to further augment the search by placing several calls on Twitter among the #FOAMed and #MedEd communities to gather additional article recommendations. Articles were screened independently by two of the authors to evaluate for any papers addressing the following three themes, which were determined a priori: instructor teaching strategies, clinical teaching models, and incorporation of technology. We included articles if either author recommended inclusion.

The search yielded a total of 2,514 articles, of which 123 were deemed to be directly relevant for inclusion in this review. When supporting data were not available, recommendations were made based upon the authors’ combined experience and consensus opinion. The level and grade of evidence was provided for each best practice statement according to the Oxford Centre for Evidence-Based Medicine criteria (Tables 1 and 2).^17^ Prior to submission, the manuscript was reviewed by the entire CORD Best Practices Subcommittee. It was subsequently posted to the CORD website for two weeks for review and feedback from the entire CORD community.

## INSTRUCTOR TEACHING STRATEGIES

### 1. Preparation

As in most areas in life, preparation is the key to success in clinical and bedside teaching. Adequate planning and preparation by the instructor, learner, and even the patient will result in a much more effective learning experience for all involved.^18^ Preparing for didactic teaching, development of teaching scripts, and review of physical examination skills prior to a shift can help alleviate instructor uncertainty and improve instructor confidence.^5^,^6^,^18^–^22^

Educators should consider priming the learner for the anticipated shift. Prior to the beginning of each teaching shift, the instructor should work with the learner to set clear expectations and goals.^3^ This includes orienting the learner to the plan for clinical teaching, getting buy-in from the learner, and setting relevant and achievable learning objectives by aligning the instructor’s and learner’s goals.^3^, ^18^–^21^, ^23^–^29^

Patients are integral to bedside teaching by delivering a unique perspective into their illness and educating learners about their disease course. The incorporation of patients into clinical teaching adds a level of complexity for preparation and planning.^18^ For bedside teaching, the instructor should help prepare the patient and teaching team. This should be done by setting expectations for the interaction with the patient, such as maintaining a respectful and professional tone, avoiding medical jargon, and involving the patient and his or her family.^30^,^31^ When incorporating a patient into bedside teaching, one should seek the patient’s permission first and set expectations prior to the encounter.^6^,^20^,^21^,^23^,^25^,^26^,^30^,^32^,^33^ However, care must be taken not to create a blind spot in clinical teaching by only focusing on specific sets of patients while avoiding others (e.g., those with communicable diseases or those deemed “difficult” patients).^33^ Patients’ autonomy should be respected at all times and they should be explicitly encouraged to ask questions, clarify or amend data, and to provide feedback to their medical team.^34^,^35^

### 2. Instructor Characteristics

Trust and support are important in clinical teaching. Establishing a collegial and supportive teacher-learner relationship is essential to create a culture that promotes effective knowledge acquisition, professional growth, and lifelong learning habits.^20^,^27^,^36^–^38^ Learners have a tendency to mirror the behavior of instructors they feel are professional and competent. In knowing this, instructors should demonstrate empathy and compassion, teaching both medical knowledge and professionalism skills. Learners value instructors who can push them to their zone of proximal development (the difference between what a learner can do without help and what they can do with help) while maintaining a safe learning environment.^19^,^20^,^30^,^39^–^41^ To help achieve this, educators should avoid “read my mind” questions.^19^,^42^ If a learner is struggling with a question, it can be beneficial to ask whether they understand the question at hand or if it was too ambiguous. An appropriate balance should be maintained between autonomy and supervision to help foster a supportive relationship with the learner while providing an opportunity for growth.^40^,^43^,^44^ Additionally, learners appreciate a positive attitude and enthusiasm for teaching, as well as candor from teachers about their own knowledge deficits.^18^,^27^,^45^,^46^ Table 3 provides a summary of qualities considered by learners to be essential in an effective clinical teacher.^18^,^19^,^31^,^47^,^48^

### 3. Knowledge Integration Strategies

Learners, while very eager and enthusiastic, may struggle with knowledge integration and retention. As an instructor, it is important to be cognizant of barriers to learning and how to overcome them. Several theories and strategies can be applied to clinical practice to help with knowledge acquisition and retention.

#### A. Cognitive Load Theory

The theory that the human brain can process only a finite amount of information at one given time, creating a bottleneck effect for learning is known as cognitive load theory.^49^–^51^ When the cognitive load is exceeded, learning and performance are both impaired. This can be avoided by selecting relevant teaching pearls that correspond to your learner’s level while avoiding teaching too much information at one time. In addition to the quantity of knowledge, educators should strive to reduce extraneous load. Extraneous load is the part of the working memory that engages in work that is not crucial to completing the learning task.^50^–^53^ Another technique in the clinical realm is to reduce extraneous activities to let the resident or student focus more on specific tasks.

#### B. Interleaving

Interleaving is when the learner alternates between semi-related topics rather than exclusively focusing on a single area for an extended period of time.^54^,^55^ One practical clinical application of interleaving would be for the learner to first see a patient with shortness of breath who has congestive heart failure, followed by a patient with shortness of breath due to chronic obstructive pulmonary disease. The learner can then compare and contrast the two different presentations.

#### C. Spaced Repetition/Retrieval Practice

Spaced repetition is when the learner spreads out studying or recall of information over time to enhance retrieval and retention.^54^,^55^ Retrieval practice is another strategy focused on knowledge retention, wherein the learner is asked to bring learned information from long-term memory back into use.^54^ One example using this concept is having the learner reiterate teaching points later in the shift, at sign out, or even on a subsequent shift to help space the acquired knowledge and encourage recall of the information at a later time. Technology, in the form of flash card programs and applications (apps) (e.g., Quizlet, Anki, Flashcard Machine, Study Blue, Study Stack) or in email form can also serve to remind the learner of information at a later date.

#### D. Importance of “Wait-time” and “Think-time”

It is important to allow adequate time for the learner to process and recall information. When teaching, an instructor typically poses a question and then waits for the learner to reply, known as “wait time 1.” The time after the learner response is known as “wait time 2.” After proposing a question to a learner, previous studies have shown that an instructor typically waits an average of only 1.5 seconds prior to interjecting the answer.^56^,^57^ However, studies have found that waiting three seconds or longer (especially after “wait time 2”) allowed the learner time to process the question and decreased failure-to-respond rates, increased perception of caring (thereby encouraging the learner to engage more actively), and increased the total number of responses received.^56^,^57^

### 4. Environmental and Timing Considerations

Clinical teaching should never hinder or delay care for patients, especially the critically ill; safety and the oath to “do no harm” take precedence over educational benefit.^41^ An instructor should select an appropriate “moment” for clinical teaching while minimizing distractions and engaging learners.^6^,^23^ In a busy ED environment, it is necessary to remind learners that interruptions frequently occur based on patient care demands. The responsibility falls on both the learner and instructor to revisit any interrupted teaching interaction to complete open discussions and teaching points.^58^ In caring for an acutely ill patient, it can be highly valuable for the learner to observe how a seasoned instructor provides medical care and communicates effectively with the patient, family, and team members.^4^,^59^ After the event, the instructor should debrief to allow discussion of medical decision-making, alternatives, and possible outcomes.^3^

It is important for the instructor to be aware of the learner’s mindset and select teaching opportunities for when the learner will be most receptive.^3^ If the learner is falling behind on his or her current patients and has several new patients to see, the instructor should select a different time to provide clinical teaching. Selecting a time when the learner is more receptive and has more available learning capacity will enhance knowledge retention.^50^–^53^ Allowing time after clinical teaching to answer clarifying questions and explain the thought process of decision-making are essential for learning and retention while providing guidance on future learning.^19^,^41^

Several strategies can be used to make on-shift teaching more efficient.^6^,^32^,^60^ The teacher could ask the learner to briefly review the literature for a given illness and then teach this back to the instructor and other learners.^23^,^30^,^31^,^37^,^60^,^61^ This will instill lessons of lifelong learning, such as strategies for accessing the literature on shift, and provides an opportunity for the learner to develop advanced knowledge on a specific topic while freeing up the teacher to see the patient. However, asking the learner to review literature should not distract from the clinical experience or teaching, and care must be taken to not overuse this practice. Additionally, it is important for the teacher to set aside time for the learner to report back the information they learned on their search. Setting a time limit for the team (e.g., less than five minutes) to keep the teaching session brief will support the learning environment without compromising ED throughput.^4^,^25^ Additionally, this ensures that teaching points remain brief, thereby avoiding the tendency to over-teach (i.e., covering an excessive amount of material in a short time span).^50^,^51^ The use of a dedicated teaching shift to protect the teacher and learner from tasks and duties that may distract from an instructional goal is another effective strategy to optimize the time available for teaching in the clinical environment.^63^

### 5. Interprofessional Considerations

Medicine has placed an increasing emphasis on the importance of interprofessional teams for the delivery of safe, efficient, cost-effective, and patient-centered care. Studies have found that non-physician colleagues who are actively involved in bedside teaching can help to improve communication around the care plan, enhance provider satisfaction with communication, reduce errors, aid in the diagnosis, shorten hospital length of stay, and reduce total hospital charges.^18^,^26^,^64^ Even if other providers (e.g., nurses, technicians, and pharmacists) are unable to be physically present at the bedside, securing buy-in from interprofessional providers and institutions for the importance of clinical teaching can minimize distractions during clinical teaching, and improve the learning experience for all involved.^6^,^25^,^65^

**BEST PRACTICE RECOMMENDATIONS**

1. Adequate preparation is crucial to the success of clinical teaching. This includes setting clear expectations, priming the learner, and seeking patient permission prior to bedside teaching (Level 2b, Grade B).
2. Learners will emulate behaviors of physicians they perceive as competent and professional. Instructors should capitalize on teachable moments and model efficient bedside history and examination skills, communication styles, respect, compassion, and humanism (Level 2b, Grade B).
3. Consider reducing cognitive load, interleaving, using spaced/retrieval practice, and increasing wait times after asking questions to allow the learner time to process and respond (Level 2a, Grade B).
4. During critically ill patient encounters, allow time to debrief after the event. Also, consider incorporating short bedside teaching points during a patient’s evaluation (Level 5, Grade D).
5. Incorporate additional members of the care team (e.g., nurses, pharmacists, technicians) into clinical teaching encounters (Level 2b, Grade B).

## CLINICAL TEACHING MODELS

It is important to use a variety of teaching strategies in the ED and tailor them to the individual learner and situation. Being creative and innovative with teaching techniques ensures that the sessions are memorable and meaningful for learners.^28^ We highlight several, well-described teaching models and describe how they can be used in the ED including the Socratic method, Aunt Minnie, the One-Minute Preceptor (OMP), SNAPPS, ED STAT, teaching scripts, and bedside presentation/rounds. Table 4 includes a summary of each of these teaching techniques with a description and example of how to implement them clinically. Additional resources for those interested in learning more are available in the Appendix.

### A. Socratic Method

In the Socratic method, the instructor poses a series of questions to a learner. One recent study found that this was the most frequently used teaching method in the ED and was used more often among higher-acuity patients, with more senior residents, and when multiple learners were present.^66^ In contrast, “pimping” (as it has been colloquially known) is an alternate approach dating back to 17th century London and is frequently confused with the Socratic method.^67^,^68^ The difference lies in the intent of the instructor toward the learner.^68^ While the Socratic method is a well-established model for improving learning and recall, pimping has a less desirable intent. It is often viewed as a “sport” aimed at reinforcing the power dynamic and hierarchy of medical training.^69^–^73^ Using increasingly difficult questions until the learner is unable to answer, the teacher shames or embarrasses the learner. Not surprisingly, this tactic impairs the trust relationship and inhibits learning.

Questioning, in general, as a teaching method has been found to be very efficient and effective.^54^,^56^,^57^,^74^ Students have been shown to better recall knowledge if it is taught after asking a question.^75^ Using this technique, advanced learners can be challenged while still teaching novices by targeting teaching and communication to meet the learner’s specific needs.^23^,^27^,^30^,^36^,^37^,^40^,^42^,^44^,^60^,^67^,^76^ To determine a learner’s existing knowledge, skills, and gaps, teachers can use probing questions (e.g., “why?” and “how would you approach…?”) to guide individualized, specific teaching to the learner, regardless of his or her level of training.^4^,^18^,^19^,^23^,^28^,^41^ Low-level questions can be used to assess factual recall, while higher-level questions assess problem-solving skills, analysis, and synthesis of the information.^24^,^30^,^32^,^34^,^36^,^37^,^40^,^42^,^43^ It is important to push a learner from basic knowledge into critical thinking and problem-solving skills through questioning. One strategy to help improve learning when approaching less familiar topics is to provide basic starting points to create the scaffolding for further problem-solving. However, when using questions as part of clinical teaching, it is essential that the learner feel safe to answer incorrectly with an emphasis on learning rather than “correct answers.”^68^

Three types of questioning have been found to be the most effective for learning: broadening, targeting, and up-the-ladder.^44^ Broadening involves asking “what if” scenarios to add educational examples beyond the current case. Targeting is the practice of asking specific questions to specific team members. The up-the-ladder technique (also referred to as “step-up questioning”) occurs when the teacher asks the same question to progressively more advanced learners. An advantage of the up-the-ladder technique is that it respects the educational advancement order and avoids the challenges of having a junior learner respond once a more senior learner has answered incorrectly.^77^

When using the Socratic method, it is important to identify the avoidant learner and gently draw him or her into the discussion. This may be facilitated by beginning with simple questions or those that you previously have confirmed the learner is able to answer correctly. While it is important to incorporate evidence-based medicine into teaching, questions that are overly advanced or not familiar to the team should be minimized as they have been shown to be less effective.^40^,^44^,^78^

Finally, lowering the stakes of the Socratic method may be accomplished by incorporating humor, explicitly stating expectations, and refraining from ego-driven discussions. The emphasis should be placed on positive reinforcement and framing questions as “learning opportunities.”^77^ Trainees should be reminded that more can be learned from incorrect answers than correct ones, as incorrect answers shed light into the learner’s knowledge gaps. The Socratic method is frequently combined with many of the techniques that follow to enhance learning and retention.

### B. Aunt Minnie

In the ED, many diagnoses occur through pattern recognition by aligning the history and physical examination with prior experiences and expertise. The “Aunt Minnie” approach is a teaching method focused on learning pattern recognition or heuristics for facilitating diagnostic efficiency. This is ideal for typical presentations of common, low-to-moderate acuity clinical complaints and allows learners to increase their repository of patient experiences as they develop their clinical gestalt. This strategy is based on the principle that, “if the lady across the street walks like your Aunt Minnie and dresses like your Aunt Minnie, she probably is your Aunt Minnie, even if you cannot identify her face.”^79^ On a deeper level, this is informed by the concept of System 1 (e.g., unconscious, automatic) and System 2 (e.g., slow, effortful) thinking.^80^ This method can be used in the ED to efficiently balance clinical care while incorporating clinical teaching of learners.^79^,^81^

For an instructor, it is important to recognize when this technique is appropriate (e.g., common ambulatory complaints) and when the model should not be used (e.g., rare or complex diseases).^82^,^83^ In the latter, learners may need to use a more strategic approach (i.e., System 2 thinking).^82^,^83^ This also provides an opportunity for educators to teach learners how to develop their gestalt. The Aunt Minnie method relies on an instructor with a good foundation of clinical experience to help facilitate the formation of pattern-recognition skills for the learner. The instructor should not be afraid to share his or her own uncertainty and doubt with the learner in more complex cases to prevent the formation of incorrect associations.

### C. One-Minute Preceptor (OMP)

The OMP model was initially described in 1992 by Neher and colleagues as a method to efficiently balance teaching while simultaneously providing effective patient care.^84^ This model is particularly well-suited for the busy ED environment. The OMP is a learner-centered model of instruction that is based on five microskills, as described in Table 4.^58^,^84^–^88^

The OMP model has shown high satisfaction among both learners and instructors with learners preferring the OMP model over the traditional precepting model.^86^ When evaluating the OMP, instructors have stated that it was more effective and efficient than the traditional model, allowing them to provide more information in the same amount of time.^89^ Multiple studies have demonstrated that teachers using the OMP feel more confident in their ability to assess the learner’s knowledge and clinical reasoning skills.^86^,^89^,^90^

The OMP model depends on the accuracy and completeness of information gathered by the learner. With more experienced learners, such as a senior EM resident, this model may be implemented rapidly in one interaction from start to finish. With more novice learners, modifications may be necessary to allow the instructor the opportunity to assess the patient and gather any missing data. Regardless, the fundamental theme of encouraging learners to commit to a diagnosis and plan is crucial to help shape their critical thinking and decision-making skills.

### D. SNAPPS (Summarize, Narrow, Analyze, Probe, Plan, Select)

The SNAPPS model emphasizes active learning by incorporating opportunities for the learners to ask the instructor questions regarding uncertainties and alternative approaches, as well as guiding self-directed, future learning. Although faculty training and ongoing commitment is required, SNAPPS does not require significantly more time than traditional teaching.^91^,^92^ A simple refinement of the SNAPPS technique incorporates the PICO (Patient, Intervention, Comparison, and Outcome) approach to frame clinical questions to guide additional self-directed learning.^93^

Multiple studies have reported that utilization of the SNAPPS model results in numerous benefits when compared with traditional teaching and the OMP model. These benefits include increases in learner satisfaction, differential diagnosis generation, expression of clinical reasoning, active engagement with teachers, generation of teaching points, opportunities for self-directed learning, and clinical skills development.^91^,^92^,^94^–^98^

### E. ED STAT (Emergency Department Strategies for Teaching Any Time)

ED STAT is the first tool specifically designed for the complex learning environment of the ED with easy-to-follow steps, allowing incorporation of clinical teaching and feedback into a single model. This model has been shown to increase the confidence in preceptors’ teaching and is designed for educators of all experience levels and backgrounds.^99^ Aside from demonstrating an increased knowledge of teaching strategies specific to the ED, this technique has also been associated with an increased satisfaction and confidence in teaching abilities by the individual.^99^

### F. Teaching Scripts

Teaching scripts are quick, specific, previously created teaching talks designed to review common complaints seen in the ED. Having these teaching scripts prepared ahead of time allows for efficient teaching during a busy ED shift.^4^,^22^,^100^ For example, when a patient presents with possible pulmonary embolism, being able to quickly summarize the diagnostic approach with a figure and references for additional reading can reduce the educator’s workload while ensuring high-quality knowledge dissemination. Instructional content for teaching scripts can include medical knowledge, communication skills, procedural training, and time management strategies. To prevent cognitive overload, instructors should focus on one topic and limit the teaching to a short time period.^41^ While some teaching opportunities will present themselves based on a particular patient complaint, others can be created by asking learners about theoretical scenarios.^100^

### G. Bedside Presentations and Rounds

Although initially a clinical teaching approach used in inpatient medicine, bedside presentations and rounds can be incorporated into the ED environment and may prove beneficial for patient care. During bedside rounds, all team members should be introduced, and the comfort and privacy of the patient should be maintained at all times.^101^ The patient should be oriented to the goal of the clinical teaching session prior to the interaction and be informed that there may be theoretical discussions (e.g., differential diagnosis development, what-if scenarios) about their illness.^19^,^20^,^30^,^32^,^42^,^102^ Of note, some experts believe that hypothetical scenarios are best left for discussion away from the patient’s bedside to avoid confusion.^30^ Patient-centered communication (both verbal and non-verbal) should be used. As such, a body part should not be referred to as “it.” The patient should be talked to and not about, and there should be mindful physical positioning between the physician, learner, and patient.^22^,^25^,^103^

With adequate preparation, an instructor can add structure and depth to the teaching session to maximize the learning opportunity, even if presenting patient complaints are limited.^18^ Several different models of bedside rounds exist that can be adapted to the ED, including basic science rounds (focus on pathophysiology, signs, and symptoms); problem-oriented rounds (focus on prioritizing and managing the presenting problem list); and clinical skills rounds (focus on history-taking and physical examination skills).^18^

There are several benefits to having learners present to the supervising clinician at the patient’s bedside. By moving away from the computer or busy workstation, the focus shifts to the patient.^102^ Learners are able to directly observe how experienced clinicians interview, examine, reason, and communicate with patients and their families. In addition, supervising clinicians can immediately clarify presentations and physical examination findings.

However, this approach has potential challenges. Learners may feel increased pressure to present all of the facts and provide a comprehensive management plan while patients may not want more sensitive issues disclosed in group teaching sessions. Residents may also fear that answering questions incorrectly in front of their patients will jeopardize their patient-physician relationship and undermine their ability to care for that patient. To avoid this, the faculty can direct questions to learners not involved in direct care of that specific patient.^4^ Alternatively, instructors can help mitigate this by guiding learners to identify or by demonstrating a particular finding.^35^

**BEST PRACTICE RECOMMENDATIONS**

1. Use questions to engage students and residents in active learning. Combine the use of low-level questions to assess knowledge and high-level questions to assess problem-solving skills. Make sure to create a supportive, safe learning environment (Level 2b, Grade B).
2. Consider OMP to promote a learner-centered model of instruction. This tool is well-received by both learners and instructors due to its focus on five microskills while incorporating feedback (Level 2b, Grade B).
3. Consider using SNAPPS to promote active participation and engagement for both learners and educators (Level 1b, Grade B).
4. Considering using ED STAT to help foster an environment of learning in the complete ED environment. It is designed for educators of all experience and backgrounds and will increase preceptors’ confidence in teaching. (Level 2b, Grade B)
5. Prepare ahead by having brief, specific, pre-created teaching scripts designed to review common ED complaints to allow for efficient teaching during a busy ED shift (Level 2b, Grade B).
6. For bedside presentations, always orient the patient to expectations prior to the interaction. Use patient-centered communication, while being cautious with hypothetical situations in front of the patient, to facilitate a successful experience (Level 2b, Grade B).

## INCORPORATING TECHNOLOGY

The use of technology can help promote learning. As clinical teaching continues to evolve, the use of technology and innovative bedside teaching approaches will increase. Learners who are involved in simulation rather than traditional, paper-based learning have been shown to demonstrate better retention skills.^104^ However, the use of technology is not just limited to formal didactics and can be used in a variety of formats, including just-in-time training, task trainers, in situ simulation, and telemedicine.

### A. Just-in-Time Training

The learning needs and preferences of medical student and resident learners continue to evolve. Digital natives crave immediate information and prefer the integration of technology in the learning process.^105^–^107^ One particular teaching modality, just-in-time training (JITT), incorporates both technology and immediate, high-yield information to satisfy digitally-savvy learners. JITT is a method of training where topic-specific education occurs in a focused, concise manner just prior to performing the task. The literature most commonly focuses on using short, predefined educational content, such as a video with simulation for procedural-based competency.

The advantages of JITT include minimizing training time, the ability to visualize the procedure prior to performing it, and allowing prompt return to clinical duties.^108^ As such, this is ideal for a high-volume ED setting. Additionally, JITT has demonstrated positive effects at the learner, patient, and system levels, while also generally being enjoyed by learners.^108^ JITT has previously been studied for splint application.^109^,^110^ When compared with reading textbooks, watching a brief JITT instructional video before splinting was shown to yield faster learning times and more successful splint applications.^110^ Another study assessed JITT for intraosseous needle placement and defibrillator use in a pediatric ED. JITT significantly increased comfort levels and the ability to perform the procedure independently by the trainee. Moreover, the use of a dedicated JITT room in the clinical environment is both feasible, effective, and can lead to improved resident confidence with fewer supervisor-reported procedural interventions.^108^,^111^,^112^ However, JITT may not be helpful for all types of procedures and training with some research showing conflicting success rate for certain procedures, such as pediatric intubation and infant lumbar puncture.^113^,^114^

Importantly, in the era of mounting technology and easy availability, it is vital to screen the JITT resources for quality and applicability prior to incorporation into clinical practice.^115^,^116^ One study performed a systematic search of YouTube^TM^ to assess videos focused on teaching ophthalmoscopy.^117^ Out of more than 7,000 videos, they identified 27 (0.4%) that were suitable for teaching this skill; however, none of them included all of the elements for a thorough education on ophthalmoscopy. Pre-identifying resources and having them ready for learners, rather than having to look them up and evaluate them in real time, can help ameliorate this.

### B. Simulation (including Task Trainers and In Situ Simulation)

Clinical procedures have been identified as one core area to improve the efficiency and effectiveness of critical care education, specifically given the need to balance patient safety with opportunities for learners to practice procedures.^60^ Strategies to bridge this gap include learning that uses computers, task trainers, and simulation. The use of simulation to enhance clinical teaching and learning continues to increase rapidly in the form of both in situ simulation and procedural training.

Task trainers can serve as a safe alternative for reinforcing the muscle memory necessary for many of the tasks required of an emergency physician. These can range from phantom limbs for peripheral intravenous line placement to transvenous pacing or pericardiocentesis models. Use of task trainers allows evaluation of procedural competencies, provides a safe environment for learning and fine-tuning skills, and allows for troubleshooting common errors that may occur in these high-stakes procedures without the added pressure of patient and time constraints. The learner can practice placing an ultrasound-guided peripheral line on an ultrasound phantom model prior to performing the procedure on a patient.

*In situ* simulation refers to simulation performed in the clinical care setting. Simulation offers the benefit of experiential learning in a realistic environment and can be run during any clinical shift. Simulation allows the opportunity for interdisciplinary interactions and communication training. This can range from high fidelity (e.g., mannequins) to low fidelity (e.g., mock cases in an empty patient room).^118^ Technology can facilitate these simulations by using stored images or videos, as well as a number of simulation smartphone apps.

### C. Telemedicine

Wearable platforms enable learners to view how they are perceived by patients and facilitate novel debriefing approaches when attendings are not in the room during the initial patient encounter. Google Glass is a wearable platform with a head-mounted optical display that is lightweight, voice-activated, and provides the opportunity for technology-assisted education.^119^,^120^ This platform allows audiences and learners to visualize what the operator is seeing in real time, thereby allowing multiple learners to experience an educational benefit from a single experience.^104^,^120^ This can also be used by learners to review and engage in self-reflection based on the encounter.^121^ Many of the features that clinical learners deem as important to clinical education can be accomplished using this model.^122^,^123^ However, it is important to be conscious of patient privacy and the Health Insurance Portability and Accountability Act. Moving forward, it is imperative that medical educators keep abreast of emerging educational technologies including personalized learning, mobile technologies, and learning analytics. Such technology has the potential to enhance learning and clinical competence within the clinical environment.^60^

**BEST PRACTICE RECOMMENDATIONS**

1. Use just-in-time training instructional videos to facilitate asynchronous teaching and procedural skills (Level 1b, Grade B).
2. Incorporate a variety of stimuli (eg, imaging, electrocardiograms, ultrasound videos) into clinical shifts to enhance teaching and engagement of the learner (Level 2b, Grade B).
3. Consider employing *in situ* simulation as an effective educational strategy when teaching in the clinical environment (Level 2b, Grade B).
4. Consider incorporating telemedicine and wearable platforms such as Google Glass to enhance teaching and feedback during clinical encounters (Level 2a, Grade B).

## LIMITATIONS

This review has several important limitations to consider. First, while our search methodology was comprehensive, some articles may nevertheless have been missed in the current review. We minimized the risk by reviewing all related studies in the bibliographies of included articles, reaching out to content and topic experts, undergoing pre-submission review and approval by the CORD community, and placing several calls via social media for further resources. Another limitation is the dearth of experimental studies specifically within the ED setting. When robust, ED-specific educational outcomes data were not available, we used studies from other fields and expert opinions. Thus, some proposed interventions may not be as effective in the ED setting and further studies are needed to establish their efficacy in our learning environment.

## CONCLUSION

Because clinical teaching is a critical tool in the education and development of all physician trainees, it is vital to have a strong foundation of the available techniques and methods for clinical teaching. Our work provides a critical review of the literature on clinical teaching for residency education with a focus on EM. Recommendations were given for instructor teaching considerations, clinical teaching strategies, and options for incorporating technology into clinical practice. We hope this manuscript will inform readers on strategies and techniques for successful clinical teaching.

## Supplementary Information



## Figures and Tables

**Table 1:** Oxford Centre for Evidence-Based Medicine levels of evidence.^17^

Level of evidence	Definition
1a	Systematic review of homogenous randomized control trial (RCT)
1b	Individual RCT
2a	Systematic review of homogenous cohort studies
2b	Individual cohort study or a low-quality RCT*
3a	Systematic review of homogenous case-control studies
3b	Individual case-control study**
4	Case series or low-quality cohort or case-control study***
5	Expert opinion

* <80% follow-up;

** includes survey studies;

*** studies without clearly defined study groups.

**Table 2:** Oxford Centre for Evidence-Based Medicine Grades of Recommendation.^17^

Grade of evidence	Definition
A	Consistent level 1 studies
B	Consistent level 2 or 3 studies or extrapolations* from level 1 studies
C	Level 4 studies or extrapolations* from level 2 or 3 studies
D	Level 5 evidence or troublingly inconsistent or inconclusive studies of any level

* “Extrapolations” indicate data were used in a situation that has potentially clinically important differences than the original study situation.

**Table 3:** Features of an effective clinical teacher.^18^,^19^,^31^,^47^,^48^

Quality	Example
Attitudes	Efficient Enthusiastic about medicine and teaching Good bedside manner Obviously interested Positive attitude Professional Stimulates learners to think about topics
Content Knowledge	Broad knowledge base Clinical and technical skill competence Challenges accepted dogma while admitting gaps in own factual knowledge Clinical reasoning Teaching ability
Humanistic	Can admit limitations and say “I don’t know” Compassionate and kind Concerned Fosters positive and supportive relationships with learners Outgoing and friendly Role model
Leadership skills	Clear communication Encourages active participation and team involvement Establishes rapport with the group Inclusive Respects individuals Sets goals and provides feedback Supportive
Learner-centered instructional strategies	Balance between didactics and bedside approaches Challenges learners to continue to grow and think independently Encourages learners to develop life-long learning skills

**Table 4:** Commonly described clinical teaching models.

Technique	Implementation	Pearls and Pitfalls
Socratic Method	Types of Questions: Broadening: Asking “what if…” questions and changing the details of a case to make it more interesting. Example: “How would the management change if the patient were 25 versus 75 years old?” Targeting Questions: Directing questions at specific team members based on their level of training. Example: For a student: “What are the most common bacteria that cause community-acquired pneumonia?” For a junior resident: “How do we decide if a patient with pneumonia needs to be admitted?” For a senior resident: “How do we recognize and manage complications of pneumonia?” Up-the-Ladder Questions: Ask the same question of the medical student, junior resident, and finally the senior resident if needed. Example: “In this patient with a recent variceal bleed, what treatments should we consider (student)? What do you think (junior resident)? Any additional considerations (senior resident)?”	Best with higher patient acuity and flow, as well as team teaching with learners of different levels. Avoid alienating the learner with arcane questions. Avoid material that most/all of the team is unfamiliar with.
Aunt Minnie	Pattern recognition: “If the lady across the street walks like your Aunt Minnie and dresses like your Aunt Minnie, she probably is your Aunt Minnie, even if you cannot identify her face.” Steps: The learner evaluates the patient and then presents only the chief complaint and the presumptive diagnosis. The learner begins the patient note while the teacher evaluates the patient. The teacher discusses the case with the learner, gives feedback, and discusses pattern recognition for the presentation. The teacher reviews the learner’s write-up and signs the medical record.	Best with lower patient volume and acuity, and with learners able to perform a history and physical examination in a timely manner. Efficient in teaching typical presentations in common illnesses. Avoid with rare or atypical presentations and complex cases.
One-Minute Preceptor (OMP)	Steps: Get a commitment from the learner on what they think is going on with the patient. Probe for supporting evidence to explore the learner’s understanding. Teach general rule(s) pertaining to the patient and case. Reinforce what was done correctly and provide positive feedback to the learner. Correct learner mistakes.	Best with high acuity patients and more advanced learners. Avoid in a busy ED with frequent task interruptions unless completed at the bedside.
SNAPPS	Steps: Summarize the history and physical examination. Narrow the differential diagnosis to the most important. Analyze the differential by discussing the diagnosis and probabilities. Probe the preceptor by asking questions about uncertainties and alternative approaches. Plan patient management together. Select a related clinical issue for additional self-directed learning.	Facilitates active adult learning through dialogue with the preceptor, management planning, and identifying issues for further learning. Avoid in a busy ED with frequent task interruptions unless completed at the bedside.
ED STAT	Steps: Expectations: Orient the learner to the ED, how the teacher and learner will work together, and clarify expectations. Diagnosis of the Learner: To make the teaching more relevant, determine their learning objectives. Set-Up: Use a specific patient care scenario to pose a question that will be used as the foundation for the teaching point. Teach: Focus teaching on high-yield, concise, and relevant information to the learner with generalizability to other similar patient case presentations. Assess and Give Feedback: Provide constructive and nonjudgmental feedback, include self-assessment as the foundation for preceptor feedback. Teacher Always (Role Model): Realize that the learner is always watching and implicitly learns a great deal. Be aware of verbal and non-verbal communication cues (body language). Acknowledge statements as facts or opinions.	Designed for the complex environment of the ED. Incorporates teaching and feedback into one tool. Determination of learner’s needs can help optimize clinical teaching.
Teaching Scripts	Tips: Instructors should have quick and specific teaching talks readily available to review common topics. Scripts should be short teaching points prepared ahead of time.	Avoid too much content to be covered in a concise manner. Preparation is essential.
Bedside Presentations	Tips: Set the stage for your learners, patient, and family beforehand. At the bedside, ask the patient and family to listen to the presentation first. Then provide any clarifications afterwards. Assign roles to team members such as providing feedback on presentations, entering orders, or starting the patient note while the presentation is given. Consider combining with the Socratic method, OMP, or SNAPPS at the bedside.	Best when teaching team are able to all round together. Must set expectations. Avoid medical jargon.

*ED*, emergency department; *STAT*, strategies for teaching any time.
