# Supplementary material for: Clinical Teaching: An Evidence-based Guide to Best Practices from the Council of Emergency Medicine Residency Directors
Source: West J Emerg Med. 2020 Jul 3;21(4):985–98. doi: 10.5811/westjem.2020.4.46060 (PMC7390547; doi:10.5811/westjem.2020.4.46060)
Supplement: Supplementary file 1 [file wjem-21-985.docx]

**Appendix**

| **Technique** | **Additional Resources** |
| --- | --- |
| **Socratic** | - Beckman TJ, Lee MC. Proposal for a collaborative approach to clinical teaching. Mayo Clin Proc. 2009 Apr;84(4):339-44. - Certain LK, Guarino AJ, Greenwald JL. Effective multi-level teaching techniques on attending rounds: a pilot survey and systematic review of the literature. Med Teach. 2011;33(12):e644-50. - Cunningham AS, Blatt SD, Fuller PG, Weinberger HL. The art of precepting Socrates or Aunt Minnie? Arch Pediatr Adolesc Med. 1999 Feb;153(2):114-6. - Jenkins C, Hewamana S, Brigley S. Techniques for effective teaching. British J of Hosp Med. 2007; 68(9): M150-153. - Ruesseler M, Obertacke U. Teaching in daily clinical practice: how to teach in a clinical setting. Eur J Trauma Emerg Surg. 2011; 37:313-316 - Ramani S, Leinster S. AMEE Guide no. 34: Teaching in the clinical environment. Med Teach. 2008;30(4):347-64. |
| **Aunt Minnie** | - Cayley WE Jr. Effective clinical education: strategies for teaching medical students and residents in the office. WMJ. 2011 Aug;110(4):178-81. - Cunningham AS, Blatt SD, Fuller PG, Weinberger HL. The art of precepting Socrates or Aunt Minnie? Arch Pediatr Adolesc Med. 1999 Feb;153(2):114-6. |
| **One-Minute Preceptor** | - Aagaard E, Teherani A, Irby DM. Effectiveness of the one-minute preceptor model for diagnosing the patient and the learner: proof of concept. Acad Med. 2004 Jan;79(1):42-9. - Farrell SE, Hopson LR, Wolff M, Hemphill RR, Santen SA. What's the Evidence: A Review of the One-Minute Preceptor Model of Clinical Teaching and Implications for Teaching in the Emergency Department. J Emerg Med. 2016 Sep;51(3):278-83. - Ferenchick G, Simpson D, Blackman J, DaRosa D, Dunnington G. Strategies for efficient and effective teaching in the ambulatory care setting. Acad Med. 1997 Apr;72(4):277-80. - Furney SL, Orsini AN, Orsetti KE, Stern DT, Gruppen LD, Irby DM. Teaching the one-minute preceptor. A randomized controlled trial. J Gen Intern Med. 2001 Sep;16(9):620-4. - Neher JO, Stevens NG. A five-step "microskills" model of clinical teaching. J Am Board Fam Pract. 1992 Jul-Aug;5(4):419-24. - Neher JO, Stevens NG. The one-minute preceptor: shaping the teaching conversation. Fam Med. 2003 Jun;35(6):391-3. - Seki M, Otaki J, Breugelmans R, Komoda T, Nagata-Kobayashi S, Akaishi Y, Hiramoto J, Ohno I, Harada Y, Hirayama Y, Izumi M. How do case presentation teaching methods affect learning outcomes? - SNAPPS and the One-Minute preceptor. BMC Med Educ. 2016 Jan 13;16:12. - Teherani A, O'Sullivan P, Aagaard EM, Morrison EH, Irby DM. Student perceptions of the one minute preceptor and traditional preceptor models. Med Teach. 2007 May;29(4):323-7. |
| **SNAPPS** | - [Barangard H, Afshari P, Abedi P. The effect of the SNAPPS (summarize, narrow, analyze, probe, plan, and select) method versus teacher-centered education on the clinical gynecology skills of midwifery students in Iran. J Educ Eval Health Prof. 2016;13:41.](http://paperpile.com/b/ahwmj0/BOAy) - [Kapoor A, Kapoor A, Kalraiya A, Longia S. Use of SNAPPS Model for Pediatric Outpatient Education. Indian Pediatr. 2017;54(4):288-290.](http://paperpile.com/b/ahwmj0/8p7l)[Nixon J, Wolpaw T, Schwartz A, Duffy B, Menk J, Bordage G. SNAPPS-Plus: an educational prescription for students to facilitate formulating and answering clinical questions. Acad Med. 2014;89(8):1174-1179.](http://paperpile.com/b/ahwmj0/Hu6h) - [Wolpaw TM, Wolpaw DR, Papp KK. SNAPPS: a learner-centered model for outpatient education. Acad Med. 2003;78(9):893-898.](http://paperpile.com/b/ahwmj0/tYY8) - [Wolpaw T, Papp KK, Bordage G. Using SNAPPS to facilitate the expression of clinical reasoning and uncertainties: a randomized comparison group trial. Acad Med. 2009;84(4):517-524.](http://paperpile.com/b/ahwmj0/Iu6x) - W[olpaw T, Côté L, Papp KK, Bordage G. Student uncertainties drive teaching during case presentations: more so with SNAPPS. Acad Med. 2012;87(9):1210-1217.](http://paperpile.com/b/ahwmj0/q7H1) |
| **ED STAT** | - Sherbino J, Frank J, Lee C, Bandiera G. Evaluating “ED STAT”: a novel and effective faculty development program to improve emergency department teaching. Acad Emerg Med 2006;13:1062-1069. |
| **Teaching Scripts** | - Aldeen AZ, Gisondi MA. Bedside teaching in the emergency department. Acad Emerg Med. 2006; 13(8): 860-866. - Lang VJ, O’Connor AB, Blatt A, Gracey C. Collaborative development of teaching scripts: an efficient faculty development approach for a busy clinical teaching unit? J Hosp Med. 2012; 1-5. - McGee S. A piece of my mind. Bedside teaching rounds reconsidered. JAMA. 2014 May 21;311(19):1971-2. |
| **Bedside Presentations** | - Aldeen AZ, Gisondi MA. Bedside teaching in the emergency department. Acad Emerg Med. 2006; 13(8): 860-866. - Alweshahi Y, Harley D, Cook DA. Students' perception of the characteristics of effective bedside teachers. Med Teach. 2007;29:204-209. - J[anicik RW, Fletcher KE. Teaching at the bedside: a new model. Med Teach. 2003;25(2):127-130.](http://paperpile.com/b/ahwmj0/zZsI) - LaCombe MA. On bedside teaching. Ann Intern Med. 1997;126:217-220. |

**Search Strategy**

**Embase:**

(((bedside OR 'bed side') NEAR/2 (teach* OR procedural OR knowledge)) OR (teach* NEAR/2 (interpersonal OR professionalism)) OR ((educating OR education OR teaching) NEAR/3 'point of care'))

Results: 1569

**Ovid Medline:**

(((bedside or "bed side") adj2 (teach* or procedural or knowledge)) or (teach* adj2 (interpersonal or professionalism)) or ((educating or education or teaching) adj3 "point of care")).mp.

Results: 999

**CINAHL:**

( (MH "Patient Bedside") OR "bedside teaching" OR bedside n3 teach* ) ) AND ( ( (MH "Teaching Methods") OR teach* ) )

Results: 228

**PsycINFO:**

((DE "Student Teaching" OR DE "Teaching") AND (bedside OR "point of care"))

Results: 102
